# The HGR motif is the antiangiogenic determinant of vasoinhibin: implications for a therapeutic orally active oligopeptide

**DOI:** 10.1007/s10456-021-09800-x

**Published:** 2021-06-07

**Authors:** Juan Pablo Robles, Magdalena Zamora, Lourdes Siqueiros-Marquez, Elva Adan-Castro, Gabriela Ramirez-Hernandez, Francisco Freinet Nuñez, Fernando Lopez-Casillas, Robert P. Millar, Thomas Bertsch, Gonzalo Martínez de la Escalera, Jakob Triebel, Carmen Clapp

**Affiliations:** 1 Instituto de Neurobiología, Universidad Nacional Autónoma de México (UNAM), Querétaro, México; 2 Instituto de Fisiología Celular, Universidad Nacional Autónoma de México (UNAM), México City, México; 3 Institute of Infectious Disease and Molecular Medicine, Department of Integrative Biomedical Sciences, Faculty of Health Sciences, University of Cape Town, Cape Town, 7925 South Africa; 4 Centre for Neuroendocrinology, Department of Immunology, Faculty of Health Sciences, University of Pretoria, Pretoria, South Africa; 5 Institute for Clinical Chemistry, Laboratory Medicine and Transfusion Medicine, Nuremberg General Hospital & Paracelsus Medical University, Nuremberg, Germany

**Keywords:** Vasoinhibin, 16K prolactin, Angiogenesis, Vasopermeability, Oligopeptide, Retina, Melanoma

## Abstract

The hormone prolactin acquires antiangiogenic and antivasopermeability properties after undergoing proteolytic cleavage to vasoinhibin, an endogenous prolactin fragment of 123 or more amino acids that inhibits the action of multiple proangiogenic factors. Preclinical and clinical evidence supports the therapeutic potential of vasoinhibin against angiogenesis-related diseases including diabetic retinopathy, peripartum cardiomyopathy, rheumatoid arthritis, and cancer. However, the use of vasoinhibin in the clinic has been limited by difficulties in its production. Here, we removed this barrier to using vasoinhibin as a therapeutic agent by showing that a short linear motif of just three residues (His46-Gly47-Arg48) (HGR) is the functional determinant of vasoinhibin. The HGR motif is conserved throughout evolution, its mutation led to vasoinhibin loss of function, and oligopeptides containing this sequence inhibited angiogenesis and vasopermeability with the same potency as whole vasoinhibin. Furthermore, the oral administration of an optimized cyclic retro-inverse vasoinhibin heptapeptide containing HGR inhibited melanoma tumor growth and vascularization in mice and exhibited equal or higher antiangiogenic potency than other antiangiogenic molecules currently used as anti-cancer drugs in the clinic. Finally, by unveiling the mechanism that obscures the HGR motif in prolactin, we anticipate the development of vasoinhibin-specific antibodies to solve the on-going challenge of measuring endogenous vasoinhibin levels for diagnostic and interventional purposes, the design of vasoinhibin antagonists for managing insufficient angiogenesis, and the identification of putative therapeutic proteins containing HGR.

**Supplementary Information:**

The online version contains supplementary material available at 10.1007/s10456-021-09800-x.

## Introduction

Abnormal angiogenesis underlies multiple diseases characterized by excessive or insufficient proliferation of blood vessels including cancer, vasoproliferative retinopathies, rheumatoid arthritis, diabetic ulcers, cardiovascular disease, and many others [1]. The concept of preventing disease progression by inhibiting angiogenesis has been extensively validated preclinically and clinically for the treatment of cancer and vasoproliferative retinopathies [2–4]. Inhibitors of tyrosine-kinase receptors [5] and monoclonal antibodies against VEGF [3] are the leading antiangiogenic compounds used clinically, albeit with drawbacks such as resistance, off-target effects, and toxicity [6, 7]. Also, several endogenous antiangiogenic proteins and protein fragments have been identified as potential therapeutic agents [8–10] with advantages of small size, lower immunogenicity, higher specificity, and lower risk of drug resistance [8, 11, 12]. However, some of these proteins are difficult to produce and have poor clinical performance [10, 11].

Hormones regulate blood vessel growth and function [13]. They act directly on endothelial cells or indirectly, via the production of angiogenic and antiangiogenic mediators, and operate systemically to coordinate angiogenesis with other functions throughout the body [13]. Vasoinhibin is a proteolytically generated fragment of the hormone prolactin (PRL) that inhibits the proliferation, migration, survival, and permeability of endothelial cells [13, 14]. It binds to a multicomponent complex formed by plasminogen activator inhibitor-1, urokinase plasminogen activator, and the urokinase plasminogen activator receptor on endothelial cell membranes [15] to inhibit the signaling pathways (Ras-Raf-MAPK; Ras-Tiam1-Rac1-Pak1; PI3K-Akt; and PLCɣ-IP_3_-eNOS) activated by several proangiogenic and vasopermeability factors (VEGF, bFGF, IL-1β, bradykinin) [13, 14]. Vasoinhibin is generated in the hypothalamus, the pituitary, and the target tissues defining the PRL/vasoinhibin axis [16]. Disruption of this axis can contribute to the progression of diabetic retinopathy [17, 18], retinopathy of prematurity [19], peripartum cardiomyopathy [20], pre-eclampsia [21], and rheumatoid arthritis [22]. However, translation of vasoinhibin into the clinic has been tempered by difficulties in its recombinant production [23]. We reasoned that the identification of the functional domain of vasoinhibin could lead to vasoinhibin-mimetics as alternative therapeutic molecules.

**Fig. 1:**
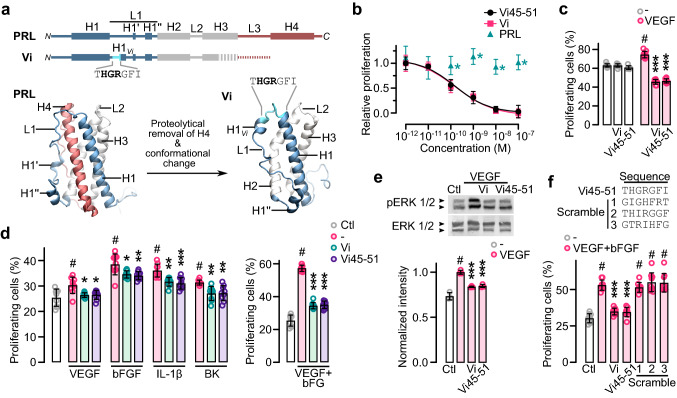
THGRGFI heptapeptide mimics vasoinhibin. **a** Diagrams of secondary and tertiary structures of prolactin (PRL) and vasoinhibin (Vi) indicating α-helixes (H1-4), loops (L1-3), and residues 45–51 corresponding to the THGRGFI. Vi originates when H4 is removed by specific proteolysis causing conformational changes in L1 including a new α-helix (H1_*Vi*_). **b** Dose–response inhibition of HUVEC proliferation by the THGRGFI heptapeptide comprising residues 45–51 of Vi (Vi45–51), Vi of 123 residues (Vi), and PRL. Inhibition was against VEGF/bFGF-induced HUVEC proliferation. Proliferation relative to those of the lowest and highest dose of Vi positive control, *n* = 9, *P < 0.001 versus Vi (Two-way ANOVA, Dunnett). Dose–response curves fitted by least square regression analysis (*r*^2^ > 0.9). Effect of 100 nM Vi or Vi45–51 on BUVEC-E6E7 basal and VEGF-stimulated proliferation (**c**), or on human umbilical vein endothelial cell (HUVEC) proliferation stimulated by the proangiogenic factors VEGF, bFGF, IL-1β, or bradykinin (BK), or the VEGF + bFGF combination (**d**). **e** Representative Western blot showing the effect of Vi or Vi45–51 on VEGF-induced phosphorylation (p) and total levels of ERK 1/2 in HUVEC and the densitometric values of phosphorylated ERK 1/2 after normalization for total ERK 1/2. **f** Effect of 100 nM Vi, Vi45–51 and three different heptapeptides with Vi residues 45–51 in random order (scramble) on the VEGF + bFGF-induced proliferation of HUVEC. Proliferating cells are expressed relative to total cells. *P < 0.033, **P < 0.002, ***P < 0.001 versus stimulated control; # *P* < 0.001 versus basal proliferation (Ctl) (Two-way ANOVA, Tukey). In all cases, values are means ± SD, *n* = 9

Vasoinhibin originates when the fourth ⍺-helix (H4) of PRL is removed by specific proteolysis within loop 3 (L3) [13, 14, 24]. The structure of vasoinhibin is unknown, but molecular dynamic simulation showed that loss of H4 induces a new conformation in the first half of loop 1 (L1) that is absent in PRL and could expose the functional determinant of vasoinhibin (Fig. 1a) [25]. In agreement, a 79-residue protein comprising the H1 and L1 regions of human PRL [25] and a 14-residue oligopeptide corresponding to the L1 sequence of buffalo PRL [26] are antiangiogenic. Here, we have identified the functional determinant of vasoinhibin and translated it into easily produced soluble oligopeptides that inhibit angiogenesis and vasopermeability with the same potency as vasoinhibin.

## Materials and methods

Materials and methods are detailed in the “Supplementary methods” section.

### Reagents

Linear peptides (> 95% pure) were commercially synthetized acetylated and amidated at the N- and C-termini, respectively (Supplementary Table S2). The cyclic-retro-inverse-vasoinhibin-(45–51)-peptide (CRIVi45–51) (> 98% pure) was synthetized with the sequence _D_Ile-_D_Phe-Gly-_D_Arg-Gly-_D_His-_D_Thr and head-to-tail cyclization (GenScript, Piscataway, NJ). Recombinant vasoinhibin of 123 residues was produced as reported [27]. Recombinant human PRL was provided by Michael E. Hodsdon [28] (Yale University, New Haven, CT). Recombinant human vascular endothelial growth factor-165 (VEGF) was a gift from Genentech (South San Francisco, CA) and basic fibroblast growth factor (bFGF) was donated by Scios, Inc. (Mountain View, CA). Bradykinin (BK) and interleukin-1β were purchased from Sigma-Aldrich (St. Louis, MO) and R&D Systems (Minneapolis, MN), respectively. Anginex peptide was acquired from Phoenix Pharmaceuticals (Burlingame, CA), and cilengitide, human angiostatin K1-3, endostatin, pazopanib, sorafenib and sunitinib from Sigma-Aldrich (St. Louis, MO). Vasoinhibin and PRL cDNA were point-mutated by the two-step PCR technique, cloned in the pcDNA3 vector and produced by HEK293T/17 (ATCC, Manassas, VA) cells as reported [23].

### Molecular dynamic simulation

Classical MD simulation was performed using GROMACS [29] using the coordinates of soluble human PRL (PDB: 1RW5) [30], as reported [25].

### Cell culture

Human umbilical vein endothelial cells (HUVEC) were obtained [31] and cultured in F12K medium supplemented with 20% fetal bovine serum (FBS), 100 μg mL^−1^ heparin (Sigma-Aldrich), and 25 μg mL^−1^ ECGS (Corning, NY). The mouse melanoma B16-F10 cell line (CRL-6475, ATCC) was cultured in DMEM medium with 10% FBS. All media contained 100 U mL^−1^ penicillin–streptomycin.

### Endothelial cell proliferation

HUVEC were seeded at 14,000 cells cm^−2^ in a 96-well plate and, after 24 h, starved with 0.5% FBS for 12 h. The medium was refreshed with 20% FBS-F12K and incubated with 100 μg mL^−1^ heparin together with 25 ng mL^−1^ VEGF and 20 ng mL^−1^ bFGF for 24 h in combination with a single dose (100 nM) or different concentrations of PRL, vasoinhibin, or synthetic oligopeptides. DNA synthesis was quantified by the DNA incorporation of the thymidine analog 5-ethynyl-2’-deoxyuridine (EdU, Sigma) labeled by the click reaction with fluorescent Azide Fluor 545 (Sigma-Aldrich) as reported [25, 32].

### ERK1/2, Akt, and eNOS phosphorylation

The protein of HUVEC cells treated or not with 100 nM recombinant vasoinhibin or Vi45–51 followed by the addition or not of 100 ng mL^−1^ VEGF, was extracted in RIPA lysis buffer supplemented with 1:100 Halt Protease-Phosphatase Inhibitor cocktail and 5 mM EDTA (both from Thermo Scientific). Proteins were resolved in SDS-PAGE followed by western blot as reported [25] with antibodies against phospho-ERK1/2 (9101, 1:500), phospho-Akt (9271, 1:500), or phospho-eNOS (9571, 1:250); and antibodies against total proteins ERK1/2 (9102, 1:500), Akt (9272, 1:500), or eNOS (9572, 1:500), all from Cell Signaling (Danvers, MA).

### Endothelial cell motility assay

The scratch wound healing assay evaluated cell motility [33]. HUVEC were grown to confluence on a 6-well plate and scratched using the edge of a cell scraper. The medium was replaced and IL-1β (10 ng mL^−1^) was added to stimulate motility with or without 100 nM of recombinant vasoinhibin or Vi45–51. After 16 h, HUVEC migration was recorded with an inverted microscope and the area of wound sealing was calculated with the CellProfiler software [34]. The wound sealing area was expressed relative to the wound area and compared against cells treated with IL-1β alone.

### Endothelial cell invasion assays

The transwell Matrigel barrier assay [35] evaluated cell invasion. HUVEC were seeded at 14,000 cells cm^−2^ on the upper (luminal) side of an 8-μm-pore insert coated with 0.38 mg mL^−1^ Matrigel (BD Biosciences, San Jose, CA) in starvation medium (0.5% FBS and no ECGS) with 100 nM recombinant vasoinhibin or Vi45–51. Conditioned medium of 3T3-L1 cells containing 10% FBS and VEGF (50 ng mL^−1^) added to the lower (abluminal) chamber served as chemoattractant. After 16 h, invading cells in the bottom side were fixed, permeabilized, stained and counted.

### Matrigel tube formation assay

Formation of tubular endothelial cell networks was evaluated by the Matrigel tube formation assay as reported [36]. HUVEC (26,500 cells cm^−2^) were seeded onto a solid layer of Matrigel (1.2 mg cm^−2^) in a 24-well plate in the presence of 200 nM recombinant vasoinhibin or Vi45–51 and incubated for 6 h. Tube formation was quantified by Angiogenesis Analyzer for ImageJ software [37].

### Vasopermeability in vitro assays

HUVEC monolayers were grown on 6.5 mm transwell with a 0.4 μm pore. Trans-endothelial electrical resistance (TEER) was measured with the epithelial EVOM^2^ Volt/Ohm meter (World Precision Instruments, Sarasota, FL). Monolayers were treated with 100 nM of recombinant vasoinhibin or Vi45–51 for 1 h before adding 50 ng mL^−1^ VEGF. TEER was measured over a 120-min period. Albumin transit across HUVEC monolayers was evaluated 10 min after VEGF addition by adding Evans blue-linked albumin into the upper chamber and measuring its transit to the lower chamber.

### Actin distribution

HUVEC cells seeded on Millicell EZ Slides (Millipore, Burlington, MA) were treated for 1 h with 100 nM recombinant vasoinhibin or Vi45–51 followed by 200 ng mL^−1^ of VEGF for 1 h. Cells were then fixed, permeabilized, and stained for actin with rhodamine phalloidin (Thermo Fisher Scientific).

### Heat and pepsin treatments

Solutions (2 μM) of recombinant vasoinhibin, Vi45–51, or CRIVI45–51 were heat-inactivated (97 °C, 15 min) or digested with pepsin (Sigma-Aldrich) by incubating 4 μM of each vasoinhibin at a final 1:20 protease to substrate molar ratio in 40 mM HCl (37 °C for 5 h).

### Animals

Male Wistar rats, and male and female C57BL6 and CD1 mice were housed under standard laboratory conditions. All interventions were carried out under 2.5% isoflurane anesthesia (SomnoSuite system, Kent Scientific, Torrington, CT). Experiments were approved by the Bioethics Committee of the Institute of Neurobiology of the National University of Mexico (UNAM) according to the US National Research Council’s Guide for the Care and Use of Laboratory Animals (Eighth Edition, National Academy Press, Washington, D.C., USA).

### Matrigel plug angiogenesis assay

The Matrigel plug assay was performed [38] and quantified [39] as reported. Matrigel plug (0.5 mL, BD Biosciences) was supplemented with 300 ng mL^−1^ bFGF and 1 μM recombinant vasoinhibin or Vi45–51.

### Retinal angiogenesis assay

Retinal angiogenesis was determined in CD1 neonate mice as reported [40]. Vi45–51 (300 μg kg^−1^) or vehicle were administered intraperitoneally every 12 h from postnatal day (P) 3 to 8. Pups were euthanized by carbon dioxide inhalation and decapitation at P8, and retinas flat-mounted or frozen to evaluate blood vessels by immunofluorescence or RT-qPCR, respectively.

### Retinal vasopermeability assay

The retinal vasopermeability assay was carried out as previously described [41]. Male Wistar rats (300 g) anesthetized with 70% ketamine and 30% xylazine (1 μL g^−1^) were intravitreally injected with 3 μL of vehicle (PBS) containing or not 200 ng of VEGF with or without 20 μM recombinant vasoinhibin or Vi45–51, and retinal vasopermeability was evaluated after 24 h by fluorescein angiography or the Evans blue method.

### Tumor model

The mouse model of primary tumor development based on the use of B16-F10 melanoma cells was used as previously reported [42]. Briefly, anesthetized female C57BL6 mice (8–12 weeks-old) were inoculated into their right flank with 10^5^ B16-F10 cells. Five days after tumor cell inoculation, mice were treated daily with vehicle (NS) or CRIVi45–51 injected into the lateral tail vein or via intragastric gavage for the 8 d following tumor cell injection. Animals were then euthanized by cervical dislocation and the tumors weighted and processed to evaluate blood vessels by immunofluorescence or RT-qPCR.

### Statistical analysis

The dose–response curves were fitted by least square regression analysis with a variable slope model calculated with at least 54 points. The unpaired t-test was used when there were two independent groups. For more than two groups One-way or Two-way ANOVA followed by the Dunnett test (when compared to a control) or Bonferroni or Tukey test (when comparing to every other group) were performed in GraphPad Prism version 8.4.3 for MacOS, GraphPad Software, San Diego, California USA. The overall significance threshold was set at *P* < 0.05.

## Results

### The HGR motif is the antiangiogenic determinant of vasoinhibin

To precisely locate the antiangiogenic determinant of vasoinhibin, we scanned synthetic oligopeptides along the 45–58 residue sequence of human PRL for their ability to inhibit endothelial cell (EC) proliferation (Supplementary Fig. 1). A heptapeptide corresponding to residues 45–51 of vasoinhibin (Vi45–51) inhibited EC proliferation with a potency (IC_50_ ~ 150 pM) and profile similar to a conventional vasoinhibin of 123 residues (Fig. 1b). Like vasoinhibin [13, 14], Vi45–51 did not affect basal EC proliferation (Fig. 1c), antagonized the EC proliferation induced by various proangiogenic factors (VEGF, bFGF, IL-1β, and bradykinin) (Fig. 1d), and prevented the VEGF-induced activation of the MAPK pathway (Fig. 1e). Moreover, three different scrambled heptapeptides failed to inhibit EC proliferation indicating that the Vi45–51 sequence is required for activity (Fig. 1f).

**Fig. 2:**
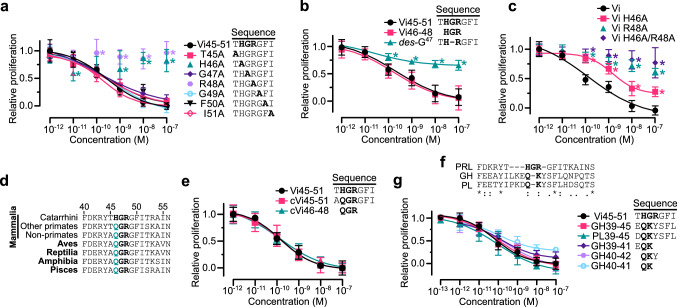
HGR motif is the antiangiogenic determinant of vasoinhibin. Dose–response inhibition of VEGF + bFGF-induced HUVEC proliferation by: Vi45–51 subjected to alanine scanning mutagenesis (**a**); by Vi45–51, HGR tripeptide (Vi46–48), and Vi45–51 with G47 deleted (*des*-G^47^) (**b**); and by vasoinhibin of 123 amino acids (Vi) or Vi alanine mutants H46A, R48A, or H46A/R48A (**c**). **d** Alignment of vertebrate PRL sequences. Conserved HGR residues in bold and substitution of H by Q in green. **e** Dose–response inhibition of VEGF + bFGF-induced HUVEC proliferation by Vi45–51, its Q-substituted conserved version (cVi45–51), and the QGR tripeptide (cVi46–48). **f** Alignment of human PRL, GH, and PL sequences indicating same (*), similar (:), less similar (.), and dissimilar (blank) residues. The HGR motif (residues 46–48) in PRL and the putative Q–K motif in GH and PL (residues 40–41) are in bold. **g** Dose–response inhibition of HUVEC proliferation by Vi45–51, heptapeptides comprising residues 39–45 of GH (GH39–45) and PL (PL39–45), EQK and QKY tripeptides (GH39–41 and GH40–42, respectively) and the QK dipeptide (GH40–41). Inhibition was against VEGF/bFGF-induced HUVEC proliferation. Values are means ± SD relative to those of the lowest and highest dose of Vi or Vi45–51 positive controls, *n* = 9, *P < 0.001 versus Vi45–51 or Vi (Two-way ANOVA, Dunnett). Dose–response curves fitted by least square regression analysis (*r*^2^ > 0.8)

The HGR motif is essential for vasoinhibin activity. Alanine-scanning revealed that H46A or R48A were the only mutations abolishing the activity of the heptapeptide (Fig. 2a), deletion of G47 (*des*-G^47^) resulted in loss of function, and the synthetic HGR tripeptide (Vi46-48) displayed full activity (Fig. 2b). Furthermore, R48A and H46A/R48A mutants of the 123-residue vasoinhibin showed no antiangiogenic activity, whereas the sole mutation of H46 to A46 resulted in a reduced effect (Fig. 2c). These findings identify HGR as the essential element responsible for the antiangiogenic activity of full-length vasoinhibin.

The essential role of HGR is further supported by its evolutionary conservation. It is conserved in the PRL of high order primates and in other vertebrates H is replaced by glutamine (Q), a conservative substitution (Fig. 2d). The conserved heptapeptide (cVi45–51) with H46Q and the QGR tripeptide (cVi46–48) displayed activity equal to Vi45–51 (Fig. 2e).

Because PRL is structurally and functionally related to growth hormone (GH) and placental lactogen (PL), which are also cleaved to vasoinhibin [13, 43], we aligned their human sequences, synthetized two heptapeptides comprising residues 39–45 of GH and PL, and showed that both of them inhibited EC proliferation with the same potency as Vi45–51 (Fig. 2f, g). Thereby, the location of the antiangiogenic domain is similar among the three hormones and, although GH and PL lack the HGR sequence, they contain Q40–K41 as a putative HGR-like motif. Like Q and H, K is a conservative substitution for R, and tripeptides EQK and QKY and dipeptide QK displayed similar activity than Vi45–51.

### Molecular mechanism encrypting the HGR motif in PRL

**Fig. 3:**
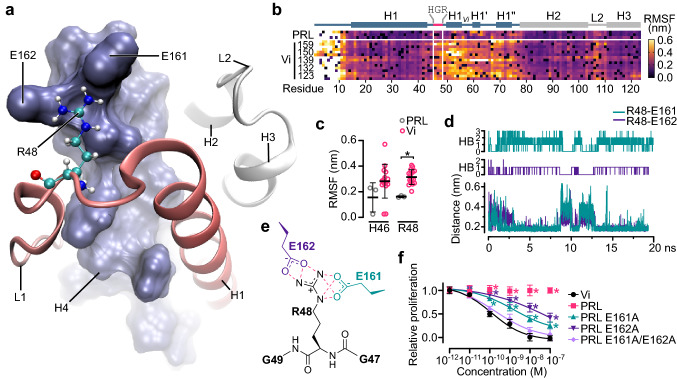
E161 and E162 in PRL obscure the HGR motif by salt bridges restraining R48. **a** Detail of the interaction between R48 (balls and sticks) and E161 and E162 in the N-terminal region of α-helix 4 (H4) (blue surface). R48 is located in loop 1 (L1) near α-helix 1 (H1) (red ribbon). **b** Root mean square fluctuation (RMSF) of the first 123 residues of PRL and vasoinhibin (Vi) of different lengths (159, 150, 139, 132, and 123 residues) over 20 ns molecular dynamic (MD) simulation. Location of HGR, α-helixes (H1-3), and loops (L1-2) are indicated in the diagram above. **c** RMSF of H46 and R48 in PRL and Vi isoforms. Values are means ± SD, *n* = 3–9, *P = 0.042 (Two-way ANOVA, Bonferroni). **d** Formation of hydrogen bonds (HB) between R48 and E161 and R48 and E162 in PRL and their correlation with the minimum distance between the residue pairs revealed by MD simulation. **e** Molecular scheme showing the three and four possible hydrogen bonds (red dashed lines) formed between the side chains of R48 and E162 and E161, respectively. **e** Dose-dependent inhibition by Vi of 123 residues (Vi), PRL, or PRL mutants where E161, E162, or E161 and E162 were replaced by alanine on VEGF + bFGF-induced proliferation of HUVEC. Values are means ± SD relative to those of the lowest and highest dose of the Vi positive control, *n* = 9, *P < 0.001 versus Vi (Two-way ANOVA, Dunnett). Dose–response curves fitted by least square regression analysis (*r*^2^ > 0.9)

The fact that PRL is not antiangiogenic [13, 14] (Fig. 1b) implies that HGR is cryptic in PRL and only exposed after removal of H4. The analysis of the HGR region in the structure of human PRL [28, 30] revealed that R48 is in contact with glutamic acids 161 and 162 (E161 and E162) (EE) located in the amino terminal region of H4 (Fig. 3a; Supplementary Fig. 2a, b). Hence, we speculated that salt bridges involving the positive charge of R48 and the negative electrostatic potential of E161 and E162 restrict the mobility of HGR preventing its activity (Supplementary Fig. 2c). Indeed, molecular dynamic simulation showed that the fluctuation of residues within L1, including H46 and R48, is higher in various vasoinhibin isoforms than in PRL (Fig. 3b, c) in which R48 forms up to 3 and 2 hydrogen bonds with E161 and E162 at minimum distance, respectively (Fig. 3d, e). Moreover, this EE motif is found in primates and rodents, and in all other vertebrates E162 is replaced by aspartic acid (D), a similar negatively charged residue (Supplementary Fig. 2d). The role of these residues in restraining antiangiogenic activity in full-length PRL is cogently supported by the demonstration that removal of these acidic side chains by alanine substitution confers antiangiogenic properties upon full-length PRL (Fig. 3f). We conclude that salt bridges between R48 and E161 and E162 obscure the HGR sequence in PRL rendering it unable to inhibit angiogenesis.

### The vasoinhibin heptapeptide (Vi45–51) containing the HGR motif inhibits angiogenesis and vasopermeability

**Fig. 4:**
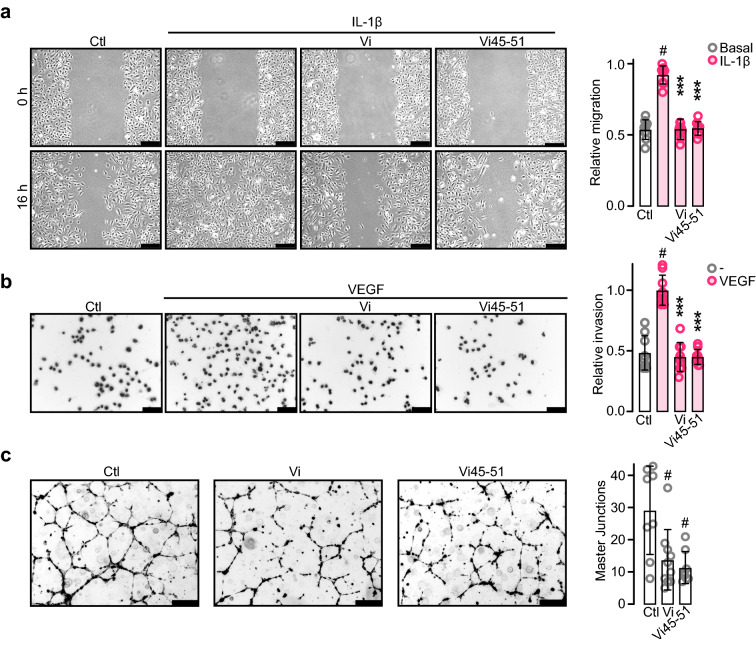
Vasoinhibin heptapeptide (Vi45–51) containing the HGR motif inhibits angiogenesis in vitro. **a** HUVEC monolayers at 0 and 16 h after wound scratch incubated in the absence (Ctl) or presence of IL-1β (10 ng mL^−1^) alone or together with 100 nM vasoinhibin of 123 residues (Vi) or Vi45–51. Values represent the area occupied by migrating cells relative to the initial wound area. **b** Inhibition of HUVEC invasion across a Matrigel barrier by 100 nM Vi or Vi45–51. VEGF (50 ng mL^−1^) was used as chemoattractant. Values are number of invading cells relative to those with VEGF alone. **c** Inhibition of HUVEC tube-network formation by 200 nM Vi or Vi45–51 quantified by number of master junctions. **P < 0.01, ***P < 0.001 versus stimulated control, # *P* < 0.01 versus Ctl. Values are means ± SD, *n* = 9, compared by Two-way ANOVA-Dunnett. Scale bar: 300 μm

**Fig. 5:**
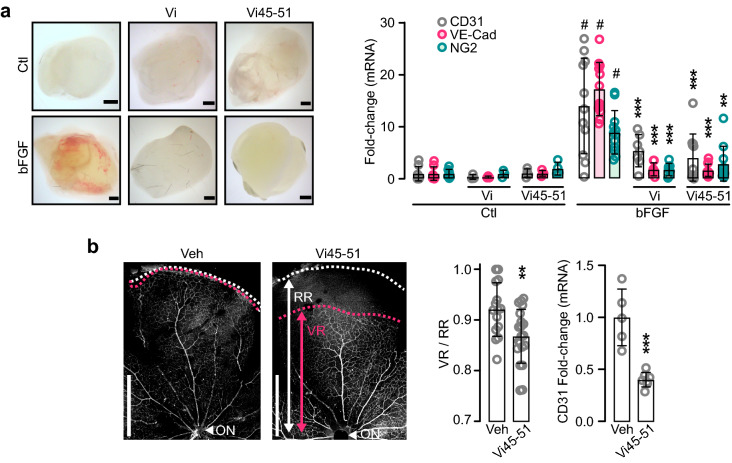
Vasoinhibin heptapeptide (Vi45–51) containing the HGR motif inhibits angiogenesis in vivo. **a** Matrigel plugs 6 d after mice implants without (Ctl) or with bFGF containing or not Vi or Vi45–51. mRNA expression of endothelial cell (CD31 and VE-Cad) and pericyte (NG2) markers in plugs. **P < 0.01, ***P < 0.001 versus bFGF; **#** *P* < 0.001 versus Ctl. **b** CD31-immunostained flat-mounted retinas from postnatal d 8 neonate mice injected with vehicle (Veh) or Vi45–51. Radial vascular expansion from the optic nerve (ON) evaluated by the index between vascular (VR) and retinal (RR) ratios and by retinal CD31 mRNA levels. **P < 0.01, ***P < 0.001 versus Veh. (Unpaired *t*-test). Values are means ± SD, *n* = 9. Scale: 1 mm (**a**), 500 μm (**b**)

Angiogenesis is a multistep process involving the proliferation, migration, and tube formation of EC, all of which are downregulated by vasoinhibin [13, 14, 24]. Consistent with HGR being the vasoinhibin antiangiogenic determinant, Vi45–51 inhibited IL-1β-induced EC motility (Fig. 4a), VEGF-induced EC invasion (Fig. 4b), and EC tube formation (Fig. 4c). Furthermore, Vi45–51 inhibited the entire process of in vivo angiogenesis. Vi45–51 blocked the bFGF-induced invasion of blood vessels into subcutaneously implanted Matrigel plugs in mice (Fig. 5a) and reduced physiological angiogenesis in the new-born mouse retina. In rodents, the retinal vasculature sprouts and spreads radially after birth completing vascularization after the first postnatal week [44]. Daily intraperitoneal injections of Vi45–51 interfered with the completion of retinal vascularization, indicating impaired angiogenesis (Fig. 5b).

**Fig. 6:**
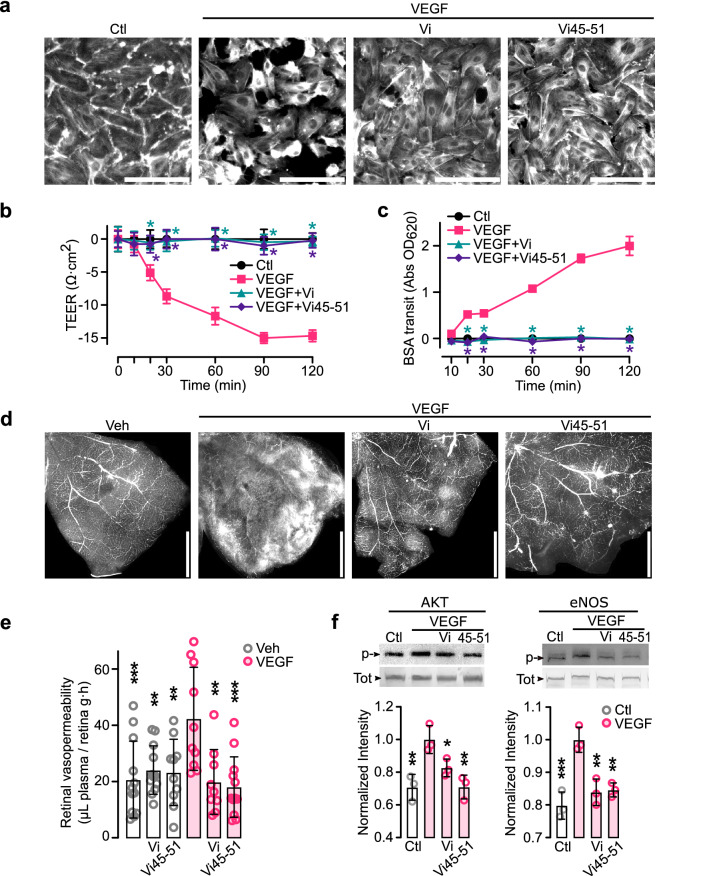
Vasoinhibin heptapeptide (Vi45–51) containing the HGR motif inhibits vasopermeability in vitro and in vivo. Effect of VEGF, or VEGF and 100 nM Vi, or VEGF and 100 nM Vi45–51 on: actin cytoskeleton distribution (200 ng mL^−1^ VEGF) (**a**), transendothelial-electrical resistance (TEER) (50 ng mL^−1^ VEGF) (**b**), and flux of Evans blue-linked albumin (BSA) (50 ng mL^−1^ VEGF) (**c**) in HUVEC monolayers. *P < 0.001 versus VEGF. Extravasation of fluorescein-labeled dextran in flat-mounted retinas (**d**) and of Evans blue-linked albumin in retinal extracts (**e**) from rats injected intravitreally with vehicle (Veh), 200 ng VEGF, or VEGF and 20 μM Vi, or VEGF and 20 μM Vi45–51. **P < 0.01, ***P < 0.001 versus VEGF. Values are means ± SD, *n* = 9, compared by Two-way ANOVA-Dunnett Scale bar: 300 μm (**a**), 1 mm (**d**)

Vasoinhibin also inhibits the excessive vascular permeability present in diseases such as diabetic macular oedema, diabetic retinopathy [41, 45, 46], and inflammatory arthritis [22]. Like vasoinhibin [45, 46], Vi45–51 blocked VEGF-induced hyperpermeability of EC monolayers as assessed by actin cytoskeleton distribution (Fig. 6a), transendothelial-electrical resistance (TEER) (Fig. 6b), and flux of Evans blue-linked albumin (Fig. 6c). Furthermore, Vi45–51 reduced the retinal extravasation of fluorescent dextran and Evans blue-linked albumin in response to the intravitreal injection of VEGF in rats (Fig. 6d, e). Vasoinhibin inhibits hyperpermeability by blocking eNOS activation and the production and effects of NO on adherent junctions [45, 46], and Vi45–51 also blocked the VEGF-induced phosphorylation/activation of AKT and eNOS (Fig. 6f). Taken together our findings indicate that HGR is the common structural element responsible for vasoinhibin vascular actions, and that oligopeptides containing HGR hold promise for the treatment of angiogenesis- and vascular permeability-dependent diseases. However, the comparison between in vitro–in vivo data provides no information regarding the relevance of a therapeutic intervention, unless pharmacokinetic and pharmacodynamic parameters establish the link between both assays.

### Oral administration of a cyclic retro-inverse vasoinhibin heptapeptide containing the HGR motif reduces tumor growth and vascularization

**Fig. 7:**
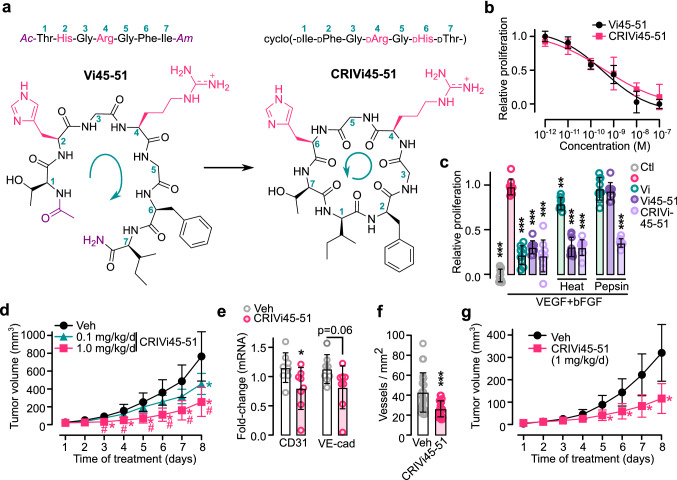
Oral administration of the cyclic retro-inverse vasoinhibin heptapeptide (CRIVi45–51) reduces tumor growth and vascularization. **a** Vi45–51 is composed of L-amino acids and acetylated (Ac) and amidated (Am) at the N- and C-termini, respectively. CRIVi45–51 is composed of D-amino acid in reverse order. Numbers indicate the ⍺-carbons and green arrows the synthesis sense. Note the conserved configuration of HGR side chains (magenta) in the two heptapeptides. **b** Dose-dependent inhibition of VEGF + bFGF-induced HUVEC proliferation by Vi45–51 or CRIVi45–51. **c** Inhibition of VEGF + bFGF-induced HUVEC proliferation by 10 nM vasoinhibin of 123 residues (Vi), Vi45–51, or CRIVi45–51 before or after heat inactivation or pepsin incubation. **P < 0.01, ***P < 0.001 versus VEGF + bFGF alone. **d** Growth curves of B16–F10 tumors in mice intravenously injected with vehicle (Veh) or with different doses of CRIVi45–51 after tumor appearance. *P < 0.002 versus Veh, #*P* < 0.002 versus 0.1 mg kg^−1^ d^−1^. mRNA expression of endothelial cell markers (CD31 and VE-Cad) (**e**) and vascular density (**f**) in tumors. *P < 0.05, ***P < 0.001 versus Veh. **g** Growth curves of B16–F10 tumors in mice after oral administration of vehicle (Veh) or CRIVi45–51 after tumor appearance. *P < 0.001 versus Veh. All values are mean ± SD, *n* = 9

Peptides have limited therapeutic application due to reduced stability and oral bioavailability [12] but substitutions with D-amino acids and cyclization can overcome these limitations [47]. Vi45–51 was optimized into a cyclic retro-inverse peptide (CRIVi45–51) composed of D-amino acids assembled in the reverse order of their native sequence to conserve the configuration of the side chains while conferring resistance to proteolysis (Fig. 7a). CRIVi45–51 inhibited EC proliferation with a similar potency to that of Vi45–51; both peptides retained full activity after heat inactivation, but only CRIVi45–51 was resistant to pepsin degradation (Fig. 7b, c).

**Fig. 8:**
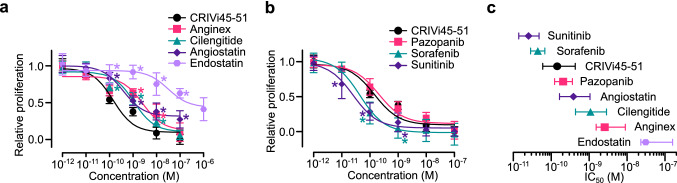
Potency of the cyclic retro-inverse vasoinhibin heptapeptide (CRIVi45–51) compared to that of other anti-cancer and antiangiogenic drugs. Dose–response inhibition of VEGF + bFGF-induced HUVEC proliferation (**a**, **b**) and respective IC_50_ values (**c**) of CRIVi45–51, antiangiogenic proteins (angiostatin and endostatin), peptides (anginex, cilengitide), and tyrosine-kinase inhibitors (pazopanib, sorafenib, sunitinib). *P < 0.001 versus CRIVi45–51. HUVEC proliferation relative to that of the lowest and highest dose of the CRIVi45–51 positive control. Dose–response curves fitted by least square regression analysis (*r*^2^ < 0.7). All values are mean ± SD, *n* = 9

Inhibition of angiogenesis by vasoinhibin suppresses the growth of colon, prostate, and melanoma tumors [42, 48, 49]. In view of its antiangiogenic properties and greater stability, CRIVi45–51 is an appealing anti-cancer compound. C57BL6 mice were intradermally implanted with B16–F10 melanoma cells and, after tumor appearance, subjected to daily intravenous injections of CRIVi45–51. CRIVi45–51 reduced tumor growth in a dose-related manner, and this effect (Fig. 7d) was associated with reduced angiogenesis. CRIVi45–51 did not inhibit the growth of melanoma cells in vitro (Supplementary Fig. 3), but it did reduce tumor vascular density and expression of CD31 (Fig. 7e, f). Notably, the oral administration of CRIVi45–51 decreased tumor growth with similar efficacy to intravenous delivery (Fig. 7g), indicating improved pharmacological properties. Furthermore, CRIVi45–51 demonstrated a greater potency in inhibiting EC proliferation than other antiangiogenic proteins (endostatin and angiostatin) and peptides (anginex and cilengitide) already tested in cancer clinical trials [11]; its potency is similar to the antiangiogenic tyrosine-kinase inhibitor pazopanib, and lower than the multi-targeted tyrosine-kinase inhibitors sunitinib and sorafenib discontinued from cancer trials due to excessive toxicity (Fig. 8a–c; Supplementary Table 1) [50].

## Discussion

Vasoinhibin is a naturally occurring angiogenesis and vasopermeability inhibitor that holds promise for targeting abnormal growth of the vasculature. A major obstacle for vasoinhibin therapeutic application has been the lack of sufficient functional protein. Post-translational modifications, protein folding, instability, and aggregation complicate the recombinant production of vasoinhibin [23]. Gene therapy vectors have allowed the efficient delivery of vasoinhibin in animal studies [41, 42] but are distant from clinical translation. Here, we show that the antiangiogenic and antivasopermeability activities of vasoinhibin reside in a short linear motif of just 3 amino acid residues (HGR) located in the early portion of L1 that upon mutation leads to vasoinhibin loss of function. Moreover, oligopeptides of 3–7 amino acids containing HGR are as potent as whole vasoinhibin, easy to produce, stable, and orally active for targeting angiogenesis and hyperpermeability in the retina and melanoma tumor vascularization and growth.

The antiangiogenic determinant of vasoinhibin has been a controversial topic. Consistent with our findings, a peptide of 14 amino acids located in the early part of L1 of buffalo PRL, unveiled due to its homology with antiangiogenic human somatostatin, exhibited antiangiogenic properties [26]. However, other work located the activity of vasoinhibin within ⍺-helix 2 (H2), specifically in a 14 amino acid sequence with “tilted peptide” characteristics [51]. This tilted sequence is highly hydrophobic and, when synthesized fused to maltose-binding protein to improve solubility, it did inhibit angiogenesis but with a potency 4- to 32-fold lower than that of vasoinhibin. Moreover, mutation to abolish tilted sequence characteristics only resulted in a partial loss of vasoinhibin function [51].

The fact that full-length PRL is devoid of antiangiogenic properties implies that the HGR motif is not active in full-length PRL. Indeed, we found that exposure of HGR is restricted in PRL due to the formation of salt bridges between R48 and residues E161 and E162 located in H4. We demonstrated the restrictive action of this interaction by showing that alanine substitution of E161 and E162 confers antiangiogenic properties upon PRL. These findings clarify the precise mechanism blocking the antiangiogenic actions in PRL. Notably, the HGR and EE motifs are highly conserved, indicating their convergent evolution to control the PRL effect on angiogenesis and implying that vasoinhibin inhibits blood vessels not only in mammals but also in lower vertebrates [52].

Vasoinhibin originating from PRL comprises a family of isoforms ranging from the first 80 to the first 159 amino acid residues, depending on the site of cleavage of different proteases (cathepsin D, matrix metalloproteinases, bone morphogenetic protein 1) [14, 16, 24]. The fact that a short linear motif of only 3 residues is the bioactive determinant suggests that all vasoinhibin isoforms share the same potency. Indeed, vasoinhibin isoforms derived from PRL, GH, and PL are equally potent [43]. The amino acid sequence of PL and GH is remarkably alike (86% homology), whereas human PRL only shares 25% sequence homology with the other two hormones [13]. The location of the antiangiogenic motif is similar among the three hormones, albeit in the GH- and PL-derived vasoinhibin isoforms this motif comprises only 2 amino acid residues (QK).

The vasoinhibin domain is an example of short linear motifs (SLiM). SLiM are typically between 3 and 10 amino acids, of which usually just two or three are important for function [53]. SLiM are very common. The updated (2018) SLiM database includes > 1 million minimotifs in > 180,000 unique proteins [54]. In particular, the HGR and QGR motifs occur in some proteins, including antiangiogenic collagen-derived peptides, such as pentastatin-3 and tetrastatin [55], whereas the QK is much more common [55, 56]. However, these motifs have not been previously validated experimentally or causally related to angiogenesis. As in vasoinhibin precursor molecules, these SLiM may be under tight regulation, so that their presence may or not confer antiangiogenic properties. For example, the SH3 binding motif RxPxxP occurs in 1 out of 20 randomly selected proteins, of which only few are functional [53]. Whether or not SLiM are functional would depend on context, including cellular compartmentalization, temporal expression, or, as revealed by our study, post-translational modifications (proteolysis, glycosylation, phosphorylation).

Unveiling the short linear motif of vasoinhibin and its evolutionary conservation has many relevant implications. First, the motif can be explored for drug development, as only few structural modifications would be required to generate soluble agonists and antagonists that are easy to produce and have low toxicity and improved pharmacokinetic properties. Second, the limited number of residues of the functional motif could help identify vasoinhibin binding partners in cell signaling processes and post-translational modifications affecting their properties. Third, the motif could impact the development of antibodies able to discriminate between vasoinhibin and PRL for quantifying vasoinhibin levels in the clinic. Finally, mutations of the vasoinhibin functional motif could be linked to several diseases in humans.

Here, we show that a cyclic retro-inverse heptapeptide is an optimized vasoinhibin analog that is resistant to pepsin degradation and orally active to inhibit vascularization and growth of melanoma tumors in mice. Furthermore, this analog inhibits endothelial cell proliferation with IC_50_ values in the pM range that are 2.7- to 214-fold lower than those of other antiangiogenic proteins (endostatin and angiostatin) and peptides (anginex and cilengitide) tested in cancer clinical trials [11]. However, significance of comparing the IC_50_ values of the various angiogenesis inhibitors is limited by the fact that they do not share receptors, co-receptors, and cell components for signaling [57].

Vasoinhibin is the subject of a completed clinical trial on peripartum cardiomyopathy (ClinicalTrial.gov, NCT00998556) and an on-going trial on diabetic retinopathy and diabetic macular oedema (ClinicalTrial.gov, NCT03161652). These trials used dopamine D2 receptor agonist- or antagonist-medications causing hypoprolactinemia or hyperprolactinemia to indirectly downregulate [58] or upregulate [59] vasoinhibin levels, respectively. The current study introduces a potent vasoinhibin analog that is small, stable, orally effective, and easy to produce for the direct clinical treatment of the above diseases and other angiogenesis-related disorders. As vasoinhibin is a broadly acting natural protein [13, 14, 16], vasoinhibin analogs could counteract the action of several proangiogenic mediators with limited resistance, side effects, and toxicity. The anticipated development of antagonists may increase clinical options for managing insufficient angiogenesis and abnormal vascular regression. Furthermore, antibodies able to discriminate between vasoinhibin and PRL may be developed to solve the on-going challenge of measuring endogenous vasoinhibin levels for diagnostic and interventional purposes.

In conclusion, our findings remove the barrier to using vasoinhibin as a therapeutic agent and provide tools for guiding research into the molecular and physiopathological actions of vasoinhibin and other proteins containing the HGR and related motifs.

## Supplementary Information

Below is the link to the electronic supplementary material.

Supplementary file1 (DOCX 3046 kb)

## Data Availability

All data generated or analyzed during this study are included in this published article and its supplementary information files.
