# Supplementary material for: The HGR motif is the antiangiogenic determinant of vasoinhibin: implications for a therapeutic orally active oligopeptide
Source: Angiogenesis. 2021 Jun 7;25(1):57–70. doi: 10.1007/s10456-021-09800-x (PMC8813873; doi:10.1007/s10456-021-09800-x)
Supplement: Supplementary file 1 — Supplementary file1 (DOCX 3046 kb) [file 10456_2021_9800_MOESM1_ESM.docx]

**Supplementary information**

The HGR motif is the antiangiogenic determinant of vasoinhibin: Implications for a therapeutic orally active oligopeptide

Juan Pablo Robles, Magdalena Zamora, Lourdes Siqueiros-Marquez, Elva Adan-Castro, Gabriela Ramirez-Hernandez, Francisco Freinet Nuñez, Fernando Lopez-Casillas, Robert P. Millar, Thomas Bertsch, Gonzalo Martinez de la Escalera, Jakob Triebel & Carmen Clapp*

*Correspondence to Carmen Clapp, **Email:** [clapp@unam.mx](mailto:clapp@unam.mx)

**This PDF file includes:**

Figures S1 to S3

Tables S1 to S2

Supplementary Experimental Procedures

SI References

**Supplementary Figures**

**Fig. S1 The heptapeptide comprising residues 45 to 51 of vasoinhibin (Vi45-51) inhibits endothelial cell proliferation with a potency similar to full-length vasoinhibin.** **a** Diagram of the secondary structure of vasoinhibin showing ⍺-helixes 1 to 3 (H1-3) and residues 40 to 65 in loop 1 (L1). Oligopeptides synthesized within the 45 to 58 sequence are indicated. The HGR motif is highlighted. **b** Dose-dependent inhibition of VEGF-induced proliferation of the bovine umbilical vein endothelial E6E7 cell line (BUVEC-E6E7) by vasoinhibin of 123 residues (Vi) or the synthetic oligopeptides shown in (a). Proliferation is relative to values corresponding to the lowest and highest doses of the Vi positive control. **P*<0.001 versus Vi (Two-way ANOVA, Dunnett). Dose-response curves fitted by least square regression analysis (r^2^ < 0.7). Values are means ± SD, n = 9

**Fig. S2 Structural analysis and conservation of the interaction of R48 with E161 and E162 in PRL**. **a** Contacts between R48 (brown translucent surface) and E161 and E162 (blue translucent surface) revealed by the overlap of 20 minimum-energy structures of PRL (PDB 1RW5). Loops (L1-3) and ⍺-helixes (H1-3) are indicated. **b** Correlation between the distance of the center of mass of R48 and E161 and R48 and E162 in the 20 minimum-energy structures of PRL. **c** Isocontour visualization of the electrostatic potential (contour level ±1 KTe^-1^) and electric field lines (brown) of PRL indicating the location of R48, E161, and 162. **d** Alignment of PRL sequences among the five vertebrate classes. Conserved E161 and E162 are in bold, and substitution of E162 by D in green

**Fig. S3 The cyclic retro-inverse vasoinhibin heptapeptide (CRIVi45-51) reduces proliferation of HUVEC but not of B16-F10 cells.** Effect of CRIVi45-51 on HUVEC or B16-F10 cell density (cells cm^-2^) over time. Values are means ± SD, n = 9, **P*<0.001 (Two-way ANOVA, Bonferroni)

**Supplementary Tables**

**Table S1.** Non-linear best fit values of antiangiogenic drugs.

| Compound | IC_50_ (pM) | 95% CI | Span (%) | r^2^ |
| --- | --- | --- | --- | --- |
| Sunitinib | 25.45 | 13.97 – 48.06 | 93.64 | 0.8860 |
| Sorafenib | 44.64 | 28.90 – 68.42 | 106.00 | 0.9022 |
| CRIVi45-51 | 145.1 | 84.77 – 289.3 | 86.86 | 0.9174 |
| Pazopanib | 206.3 | 123.4 – 367.2 | 83.73 | 0.9156 |
| Angiostatin | 388.0 | 222.5 – 665.4 | 68.47 | 0.9024 |
| Cilengitide | 1,097 | 655.1 – 1,805 | 81.49 | 0.9176 |
| Anginex | 2,560 | 1,035 – 6,560 | 73.13 | 0.8285 |
| Endostatin | 31,150 | 8,116 – 127,900 | 49.98 | 0.6875 |

**Table S2.** Synthetic peptides.

| IUPAC Name | Alias | Sequence | Molecular mass (Da) |
| --- | --- | --- | --- |
| vasoinhibin-(45-51)-peptide | Vi45-51 | THGRGFI | 827.93 |
| vasoinhibin-(45-58)-peptide | Vi45-58 | THGRGFITKAINSC | 1545.77 |
| vasoinhibin-(47-53)-peptide | Vi47-53 | GRGFITK | 818.97 |
| vasoinhibin-(49-55)-peptide | Vi49-55 | GFITKAI | 789.97 |
| vasoinhibin-(52-58)-peptide | Vi52-58 | TKAINSC | 776.91 |
| vasoinhibin-(46-48)-peptide | Vi46-48 | HGR | 409.45 |
| Des-G^47^-vasoinhibin-(45-51)-peptide | *des*-G^47^ | THRGFI | 770.83 |
| [Ala^45^]-vasoinhibin-(45-51)-peptide | T45A | AHGRGFI | 797.91 |
| [Ala^46^]-vasoinhibin-(45-51)-peptide | H46A | TAGRGFI | 761.87 |
| [Ala^47^]-vasoinhibin-(45-51)-peptide | G47A | THARGFI | 841.96 |
| [Ala^48^]-vasoinhibin-(45-51)-peptide | R48A | THGAGFI | 742.83 |
| [Ala^49^]-vasoinhibin-(45-51)-peptide | G49A | THGRAFI | 841.96 |
| [Ala^50^]-vasoinhibin-(45-51)-peptide | F50A | THGRGAI | 751.84 |
| [Ala^51^]-vasoinhibin-(45-51)-peptide | I51A | THGRGFA | 785.85 |
| Scramble 1 | Scr1 | GIGHFRT | 827.93 |
| Scramble 2 | Scr2 | THIRGGF | 827.93 |
| Scramble 3 | Scr3 | GTRIHFG | 827.93 |
| Conserved vasoinhibin-(45-51)-peptide | cVi45-51 | AQGRGFI | 788.89 |
| Conserved vasoinhibin-(46-48)-peptide | cVi46-48 | QGR | 400.43 |
| GH-vasoinhibin-(39-45)-peptide | GH39-45 | EQKISFL | 955.07 |
| PL-vasoinhibin-(39-45)-peptide | PL39-45 | DQKISFL | 941.04 |
| GH-vasoinhibin-(39-41)-peptide | GH39-41 | EQK | 444.48 |
| GH-vasoinhibin-(40-42)-peptide | GH40-42 | QKY | 478.54 |
| GH-vasoinhibin-(40-41)-peptide | GH40-41 | QK | 315.37 |

**Supplementary Methods**

**Alignment analysis.** PRL sequences of the five vertebrate classes obtained from the NCBI Reference Sequence (RefSeq) database and the complete amino acid sequences of human PRL (NP_001157030.1), PL (NP_001308.1), and GH (NP_002050.1) were aligned by maximum similarity using MUSCLE 3.8 [1].

**Point mutations to alanine in vasoinhibin and PRL.** The human PRL cDNA, including the signal peptide, was cloned into the BamH1 and NotI sites of pcDNA3 downstream of the cytomegalovirus (CMV) promoter (pcDNA3::hPRL). Generation of vasoinhibin with point mutations was performed by two steps as follows. First, PCR fragments were generated from pcDNA3::hPRL using pCMV forward primer (5’-cccacttggcagtacatca-3’) and H46A reverse primer (5’-cccggccagcggtataccgt-3’), R48A reverse primer (5’-gtaatgaaccccgcgccatgggtata-3’), or H46A/R48A reverse primer (5’-tgaaccccgcgccagcggtataccgtttatcg-3’). Second, PCR fragments were generated using H46A forward primer (5’-tataccgctggccgggggttc-3’), R48A forward primer (5’-tatacccatggcgcggggttcattac-3’), or H46A/R48A forward primer (5’-taccgctggcgcggggttcattaccaa-3’) and 123-7His-NotI reverse primer (5’-aaaaaagcggccgcttaatgatgatgatgatgatgatgggtttgctcctcaatctctacagc-3’), which introduces a stop codon in 124 position. The two corresponding fragments of each of the three vasoinhibin constructions were PCR amplified together without primers and cloned into the BamH1 and NotI sites of pcDNA3. Point mutations to alanine in PRL were obtained using a similar procedure as follows. PCR fragments were amplified from pcDNA3::hPRL with pCMV forward primer (5’-cccacttggcagtacatca-3’) and E161A reverse primer (5’-aggcgagactctgcatcagccatc-3’), E162A reverse primer (5’-aaaggcgagacgcttcatcagccatctgc-3’), or E161A/E162A reverse primer (5’-aaaggcgagacgctgcatcagccatctg-3’). Subsequently, PCR fragments were generated using E161A forward primer (5’-gatggctgatgcagagtctcgcct-3’), E162A forward primer (5’-gatggctgatgaagcgtctcgccttt-3’), or E161A/E161A forward primer (5’-gatggctgatgcagcgtctcgccttt-3’) and the PolyA reverse primer (5’-gaatagaatgacacctactcagac). The sequence of the expression plasmids was verified by the Sanger method. The plasmids with mutated cDNAs for vasoinhibin and PRL and the non-mutated PRL plasmid (pcDNA3::hPRL) were transfected into HEK293T/17 (ATCC, Manassas, VA) cultured in DMEM medium supplemented with 10% FBS and antibiotics. The transfection mixture contained the plasmid (40 μg) and 125 mM CaCl_2_ in HBSS buffer. After 24 h the medium was refreshed with 50% of the volume and, after 48 h, conditioned media were harvested, aliquoted, and stored (-70°C). The conditioned medium from cells transfected with no plasmid served as negative control. The presence of recombinant PRL and vasoinhibin isoforms in conditioned media from transfected HEK293T/17 cells was evaluated by tricine SDS-PAGE on a 16% acrylamide gel followed by immunoblotting with polyclonal anti-human PRL antiserum (CL-1) [2] carried out as reported [3]. Detection was by chemiluminescence using the peroxidase AffiniPure donkey anti-rabbit IgG (Jackson ImmunoResearch, West Grove, PA), the SuperSignal West Pico PLUS Substrate kit (Thermo Scientific, Whaltham, MA), and the Protein simple fluorchem imager and gel documenter system (ProteinSimple, San Jose, CA). Quantity One 1-D software (BioRad, Hercules, CA) determined optical density values that were interpolated into a PRL standard curve (run within the same blot) to quantify protein concentration.

**Cell culture.** The bovine umbilical vein endothelial cell line BUVEC-E6E7 generated as reported [4] was cultured in F12K medium with 10% FBS and 100 U mL^-1^ penicillin-streptomycin.

**Endothelial cell proliferation.** BUVEC-E6E7 cells seeded at 14,000 cells cm^-2^ in a 96-well plate were starved with 0.1% FBS for 24 h and treated or not with VEGF (50 ng mL^-1^) in starvation medium with or without different concentrations of five peptides synthesized along the 45 to 58 residue sequence of human PRL or with recombinant vasoinhibin of 123 amino acids (positive control). HUVEC cells were seeded at 14,000 cells cm^-2^ in a 96-well plate for 24 h and starved with 0.5% FBS for 12 h. Medium was then replaced by 20% FBS-F12K and 100 μg mL^-1^ heparin containing or not VEGF (25 ng mL^-1^), bFGF (20 ng mL^-1^), IL-1b (10 ng mL^-1^), or BK (10 μM) or a combination of 25 ng mL^-1^ of VEGF and 20 ng mL^-1^ of bFGF for 24 h in the presence or absence of a single dose (100 nM) or different doses of PRL (negative control), recombinant vasoinhibin, Vi45-51, synthetic peptides having the same residue composition as the 45-51 segment but in scrambled order (negative control), Vi45-51 subjected to alanine scanning mutagenesis, a tripeptide comprising residues 46 to 48 of vasoinhibin (Vi46-48), a Vi45-51 where G47 was deleted (*des*-G^47^), a conserved version of the Vi45-51 (cVi45-51) and the QGR tripeptide, heptapeptides comprising residues 39 to 45 of growth hormone (GH39-45) or placental lactogen (PL39-45), tripeptides EQK and QKY, the dipeptide QK (Table S2), alanine mutants of the vasoinhibin with 123 residues (Vi-H46A, Vi-R48A, Vi-H46A/R48A), alanine mutants of PRL (PRL-E161A, PRL-E162A, PRL-E161A/E162A), CRIVi45-51, antiangiogenic proteins (endostatin, angiostatin), antiangiogenic oligopeptides (anginex, cilengitide), or antiangiogenic tyrosine-kinase inhibitors (pazopanib, sunitinib, sorafenib). DNA synthesis was quantified by the DNA incorporation of the thymidine analogue 5-ethynyl-2’-deoxyuridine (EdU, Sigma) added at the time of treatments and labelled by the click reaction with fluorescent Azide Fluor 545 (Sigma-Aldrich) as reported [3, 5]. Images were obtained in a fluorescent inverted microscope (Olympus IX51, Japan) and quantified using the CellProfiler software [6]. The number of EdU-positive cells over total DNA (Hoechst)-stained cells was expressed as percentage proliferation. B16-F10 cells and HUVEC were seeded at 12,600 cells cm^-2^ and 7,400 cells cm^-2^, respectively, in a 24-well plate and incubated for different times in complete medium in the absence or presence of 100 nM CRIVi45-51 added every 24 h. Cells were trypsinized and counted.

**ERK1/2, Akt, and eNOS phosphorylation.** HUVEC cells were cultured until 80% confluence in 100 mm culture dishes, starved (0.5% FBS without ECGS and heparin) for 3 h, and treated or not for another h with 100 nM recombinant vasoinhibin or Vi45-51 followed by the addition or not of 100 ng mL^-1^ VEGF. After 10 min, cells were washed twice with cold TBS, scraped with 200 μL RIPA buffer supplemented with 1:100 Halt Protease-Phosphatase Inhibitor cocktail and 5 mM of EDTA (both from Thermo Scientific), centrifuged (10,000 x g, 4°C for 10 min), and supernatants aliquoted and stored (-70°C) until western blot analysis. Supernatants (80 μg of protein) were blotted with antibodies against phosphorylated proteins and goat anti-rabbit horseradish peroxidase secondary antibodies (111-035-144, 1:5000, Jackson ImmunoResearch, Philadelphia, PA). Immunoblots were developed using the SuperSignal West Pico PLUS Chemiluminescent Substrate kit (Thermo Fisher Scientific) in the FluorChem E imager and gel documenter system (ProteinSimple). Membranes were then reblotted using antibodies against total proteins, goat anti-rabbit alkaline phosphatase secondary antibodies (Jackson ImmunoResearch), and a colorimetric detection kit (BioRad, cat. 1706432). Quantity One 1-D analysis software (BioRad) evaluated optical densities.

**Matrigel plug angiogenesis assay.** Matrigel plug (0.5 mL, BD Biosciences) was implanted into right and left ventral areas of male C57BL6 mice (6 weeks old). After 6 days, animals were euthanized by cervical dislocation and the Matrigel was dissected, photographed, weighed, snap-frozen in liquid nitrogen, and stored at -70°C. HEK293T cells (10^4^) were added per mg of harvested plug as an external cell tracer. mRNA levels of endothelial cell markers (CD-31 and vascular endothelial-cadherin, VE-Cad) and pericyte marker (NG2) were measured by mouse-specific RT-qPCR and data were normalized for human GAPDH mRNA levels as follows. Total RNA from the Matrigel and HEK293T cell-mix was isolated using TRIzol reagent (Invitrogen, Carlsbad, CA), retrotranscribed with the High-Capacity cDNA Reverse Transcription kit (Applied Biosystems, Foster City, CA) and quantified using Maxima SYBR Green qPCR Master Mix (Thermo Fisher Scientific) in a final reaction of 10 μL containing 20 ng of cDNA, and 0.5 μM of each of the following mouse primers: CD31 forward (5’-cggttatgatgatgtttctgga-3’) and reverse (5’-aagggaggacacttccacttct-3’); VE-Cad forward (5’-ttgggctttctgactgttgt-3’) and reverse (5’-cagggacttcgtgggttt-3’); and NG2 forward (5’-gaggtcttggtgaacttcaccc-3’) and reverse (5’-gacagtaggagaccgatggtgt-3’). The primers for human GAPDH were forward (5’-gaaggtcggagtcaacggatt-3’) and reverse (5’-tgacggtgccatggaatttg-3’). The amplification conditions were 10 s at 95°C, 30 s at each primer pair-specific annealing temperature, and 30 s at 72°C for 35 cycles. The mRNA expression levels were calculated by the 2^-ΔΔCT^ method normalized to the human GAPDH transcript.

**Retinal angiogenesis assay.** CD1 pups of either sex from postnatal day (P) 3 to 8 were intraperitoneally injected every 12 h with vehicle (PBS) or 300 μg kg^-1^ of Vi45-51. At P8 pups were euthanized by carbon dioxide inhalation and decapitation, and retinas were either processed or frozen to evaluate blood vessels by immunofluorescence or RT-qPCR, respectively. For immunofluorescence, the eyes were fixed in 4% paraformaldehyde for 10 min and the retinas were dissected, maintained in cold-methanol for at least 20 min, washed, and permeabilized and blocked with PBS containing 1% Triton X-100, 0.4% BSA, and 10% normal goat serum for 48 h at 4°C. Retinas were then immunostained for 48 h at 4oC with a 1:100 dilution of the anti-CD31 polyclonal antibody (ab28364; Abcam, Cambridge, MA), washed, and labelled overnight with a 1:500 dilution of Alexa Fluor 555 goat anti-rabbit secondary antibody (A32732; Invitrogen). Finally, retinas were washed, flat-mounted, cover-slipped using Vectashield H-1000 (Vector Laboratories, Inc., Burlingame, CA), observed and digitized under a confocal microscope (LSM 780 DUO; Carl Zeiss, Oberkochen, Germany). The vascular radius (VR, from the optic nerve to the vascular front) over the retinal radius (RR, from the optic nerve to retinal edge) was determined as index of retinal vascular expansion. Total RNA was extracted using the Qiagen RNeasy Mini Kit (Qiagen, Valencia, CA) and retrotranscribed to cDNA with the High-Capacity cDNA Reverse Transcription Kit (Applied Biosystems). CD-31 mRNA levels were quantified by RT-qPCR using Maxima SYBR Green qPCR Master Mix (Thermo Scientific) in a final reaction of 10 μL containing 20 ng of cDNA, 0.5 μM CD31 primers [forward (5’-cggttatgatgatgtttctgga-3’) and reverse (5’-aagggaggacacttccacttct-3’)], and GAPDH primers [forward (5’-gaaggtcggtgtgaacggatt-3’) and reverse (5’-tgactgtgccgttgaatttg-3’)]. The amplification conditions were 10 s at 95°C, 30 s at each primer pair-specific annealing temperature, and 30 s at 72°C for 40 cycles. The data were analyzed by the 2^-ΔΔCT^ method, and Ct values were normalized to GAPDH.

**Retinal vasopermeability assay.** Male Wistar rats (300 g) anesthetized with 70% ketamine and 30% xylazine (1 μL g^-1^) were injected intravitreally with 3 μL of vehicle (PBS) containing or not 200 ng of VEGF with or without 20 μM recombinant vasoinhibin or Vi45-51, and retinal vasopermeability was evaluated after 24 h by fluorescein angiography or the Evans blue method. Anesthetized rats were injected intrajugularly with 100 mg kg^-1^ fluorescein isothiocyanate-labelled dextran (50 mg mL^-1^, FITC-dextran, MW 2·10^6^ Da), and euthanized 1 h later. Their retinas were flat-mounted, fixed for 4 h in 4% paraformaldehyde at room temperature, washed, mounted on glass slides with Vectashield (Vector Laboratories, Burlingame, CA), and observed under a fluorescence microscope. The Evans blue tracer (45 mg kg^-1^, Sigma-Aldrich) was injected intrajugularly into anesthetized rats. Two h later, 1 mL of blood was obtained from the heart to quantify Evans blue concentration in plasma and rats were perfused via the left ventricle with 100 mL PBS (pH 3.5 at 37°C) at 50 mL min^-1^. The retinas were dissected, vacuum-dried for 4 h, weighed, incubated in 200 μL of formamide (Mallinckrodt Baker, Phillipsburg, NJ) at 72°C for 18 h, and centrifuged at 300,000 x g for 60 min at 4°C. The absorbance of supernatants was evaluated at 620 nm, and the values were interpolated using a standard curve of Evans blue in formamide normalized to the retina and body weight and to the Evans blue concentration in plasma.

**Tumor model.** Female C57BL6 mice (8-12 weeks-old) under 2.5% isoflurane anesthesia (SomnoSuite system, Kent Scientific, Torrington, CT) were inoculated into their right flank with 10^5^ B16-F10 cells in 50 μL PBS. Tumor length and width were measured daily, and volume was estimated by the formula: Volume = (length x width^2^) x 0.5. Five days after tumor cell inoculation, mice were injected daily into the lateral tail vein with vehicle (normal saline) or 0.1 or 1.0 mg kg^-1^ CRIVi45-51, and tumor volumes were assessed daily for the following 8 d. In other experiments, 1.0 mg kg^-1^ CRIVi45-51 or vehicle was delivered daily intragastrically through a 20G x 1.5’’ curved metal gavage canula (Cadence Science, Staunton, VA) for the 8 d following tumor cell injection. Animals were then euthanized by cervical dislocation and the tumors processed or frozen to evaluate blood vessels by immunofluorescence or RT-qPCR, respectively. For immunofluorescence, tumors were dissected, fixed in 4% paraformaldehyde for 48 h, cryoprotected for 24 h in 10, 20, and 30% sucrose, frozen in Tissue Freezing Medium (Leica Biosystems), cut into 5 μm cryostat sections, and mounted on SuperFrost/Plus slides (Microm, Francheville, France). Sections were permeabilized with 1% Triton X-100, blocked with 1% BSA and 0.1% Tween20 in PBS for 1 h, and immunostained overnight at 4°C with a 1:200 dilution of anti-CD31 polyclonal antibody (ab28364, Abcam) followed by detection with a 1:500 dilution of Alexa Fluor 555 goat anti-rabbit secondary antibody (A32732, Invitrogen). Tumor sections were cover-slipped with Vectashield H-1000 (Vector Laboratories) and digitalized at 20X and 40X magnification under a fluorescence microscope (Olympus BX60). The number of vessels per tumor area measured vascular density. CD-31 and VE-Cad mRNA levels were quantified by RT-qPCR following the method already described in the retinal angiogenesis assay section.

**SI References**

1. Edgar RC (2004) MUSCLE: multiple sequence alignment with high accuracy and high throughput. Nucleic Acids Res 32:1792–1797. https://doi.org/10.1093/nar/gkh340

2. Corbacho AM, Macotela Y, Nava G, et al (2000) Human umbilical vein endothelial cells express multiple prolactin isoforms. J Endocrinol 166:53–62

3. Robles JP, Zamora M, Velasco-Bolom JL, et al (2018) Vasoinhibin comprises a three-helix bundle and its antiangiogenic domain is located within the first 79 residues. Sci Rep 8:17111. https://doi.org/10.1038/s41598-018-35383-7

4. Cajero-Juarez M, Avila B, Ochoa A, et al (2002) Immortalization of bovine umbilical vein endothelial cells: a model for the study of vascular endothelium. Eur J Cell Biol 81:1–8. https://doi.org/10.1078/0171-9335-00213

5. Salic A, Mitchison TJ (2008) A chemical method for fast and sensitive detection of DNA synthesis in vivo. Proc Natl Acad Sci U S A 105:2415–2420. https://doi.org/10.1073/pnas.0712168105

6. Carpenter AE, Jones TR, Lamprecht MR, et al (2006) CellProfiler: image analysis software for identifying and quantifying cell phenotypes. Genome Biol 7:R100. https://doi.org/10.1186/gb-2006-7-10-r100
